# Molluscicidal Activity of the Methanol Extract of *Callistemon viminalis* (Sol. ex Gaertner) G.Don ex Loudon Fruits, Bark and Leaves against *Biomphalaria alexandrina* Snails

**Published:** 2014

**Authors:** Ahmed A Gohar, Galal T Maatooq, Sahar R Gadara, Walaa S Aboelmaaty, Atef M El-Shazly

**Affiliations:** a *Pharmacognosy Department, Faculty of Pharmacy, Mansoura University, Mansoura 35516, Egypt*.; b *Parasitology Department, Faculty of Medicine, Mansoura University, Mansoura 35516, Egypt.*

**Keywords:** *Callistemon viminalis*, Molluscicides, *Biomphalaria alexandrina*, Histopathology, Snails

## Abstract

Methanol extracts of *Callistemon viminalis *(Sol. Ex Gaertner) G.Don Ex Loudon fruits, bark and leaves were tested for molluscicidal activity. Snails were collected and kept in dechlorinated water under standard condition. Ten adults *Biomphalaria Alexandrina, *of the same size, were introduced in plastic acquaria for each experiment. The fruits, barks and leaves were extracted with methanol and the methanol extracts were kept for testing as molluscicides. Different extracts proved to have molluscicidal activity against the vector of schistosomiasis, *B. alexandrina * snails. LC_50 _values for *C. viminalis *fruits, bark and leaves were 6.2, 32 and 40 ppm respectively. The *C. viminalis *fruits extract showed the highest effect against the tested snails. Histopathological studies proved that the site of action of all tested extracts was localized in the digestive system and hermaphrodite gland.

## Introduction

Currently, there is an increased attention for the use of new molluscicides which are highly effective, rapidly biodegradable, less expensive, readily available and probably easily applicable with simple techniques than synthetic molluscicides. One of the new trends in the biological control of vectors of diseases is testing the toxicity of plant extracts, as alternatives to chemical molluscicides, which proved to be environmentally safe and have less residual activity. There are many restrictions of using toxic compounds (pesticides and molluscicides) with fresh water. Therefore, the safety of plant extracts to human being is an advantage for studying their effect against the snail vectors of schistosomiasis.

The botanical molluscicides are of economic importance, especially in developing countries (1). Also, there is a continuous need to search for new plant species with ideal molluscicidal properities (2-3). Different plants have been reported as molluscicides (4-7). In Egypt, screening of local plants for molluscicidal activity has received increasing attention (8-13).

The treatment of *B. alexandrina* snails with sublethal concentration of *C. lanceolatus* was effective in altering the amino acid profile of this snail species which could be contributed to the impairment of snails egg laying capacity, snail-schistosome miracidiae finding mechanisms and immune response of the molluscan hosts but has no effect on the mammalian skin penetration rate by schistosome cercariae (14). The genus *Callistemon* was reported to contain diverse chemical profile; steroids and triterpenes (15 - 18), flavonoids (15, 19 and 20), tannins and phenolic compounds (15, 19 and 21), tetradecahydroxanthenediones (22), in addition to the essential oils **(**23, 24). The diversity of chemical constitution of different Callistemon species reflects diversity in its biological activities; antibacterial and antifungal activities (25**, **26), molluscicidal activity (14), Bio-repellents for land leeches, insecticidal and anthelmintic (27- 29), in addition to antioxidant and hepatoprotective activity (21), antithrombin (30), antidiabetic (31), anti-inflammatory (32), anti- alzheimers disease (20).

Reviewing the current literatures, *C.viminalis* was not previously investigated for mulloscicidal activity. So, the aim of the present study is to evaluate the efficacy of the methanolic extracts of *C. viminalis* (Sol. Ex Gaertner) G.Don Ex Loudon fruits, bark and leaves as molluscicides against Biomphalria alexandrina snails. Some histological parameters in snails tissues were determined.

## Experimental

*Materials and methods*

*Collection of snails*

Fresh water snails were collected by using ordinary snail traps with long hand (33) from the irrigation and drainage canals in Mansoura, Dakahlia Governorate. The snails were individually isolated and placed in plastic bags with a suitable amount of water from the same source and immediately transported to the laboratory (34). The snails were identified according to Ibrahim *et al. *(35).

*Maintenance of snails*

Laboratory bred *B. alexandrina *snails were used in the work. The snails were washed several times and maintained in dechlorinated tap water to acclimatize to the laboratory conditions for four weeks before experimentation (36). The average size of the used adult snails was 10-14 mm in diameter. The snails were kept separately in plastic aquaria, 8-10 liters capacity, in dechlorinated tap water allowing 10 snails/liter to avoid crowding. The aquaria contained no mud, sand, gravel or any other substratum. Dechlorinated water was obtained by storing tap water in large containers for at least 48 hours up to one week. Water in the aquaria was changed as frequent as required to keep the snails in a good condition for experiments. Dead snails were removed every day, and no artificial aeration was used. The snails were fed on fresh boiled lettuce leaves, and they were kept under natural illumination. The temperature was the ordinary room temperature with its natural diurnal fluctuations (24-26 ± 2). Maintenance of snails was established in Parasitology Department, Faculty of Medicine, Mansoura University.

*Plant materials*

*C. viminalis *(Sol. Ex Gaertner) G.Don Ex Loudon fruits, bark and leaves. The plant identity was confirmed by Dr. Ibrahim Mashaly; Professor of systematic botany, Faculty of Science, Mansoura University, Egypt.

*Preparation of plant extracts*

The shade-dried fruits, bark and leaves (1 Kg, each) were powdered and extracted by percolation in two liters percolator with distilled methanol. The combined methanol extracts of each part were concentrated to a syrupy consistency under reduced pressure and then allowed to dry in a desiccator over anhydrous CaCl_2_. The dried extracts were kept for testing as molluscicides.

*Bioassay*

Ten adults *B. alexandrina *of the same size were introduced in plastic acquaria (25×13×7 cm) with the selected concentration of the tested extract. Three grams of each extract were transferred into 100 mL volumetric flasks, and then diluted with dechlorinated tap water to complete the volume. These solutions were used to prepare the required concentrations. Different concentrations of the tested extracts (10, 20 and 40 ppm) were used at three replicates and three containers were left as control. The results were recorded every 24 hours up to 5 days for the number of dead snails. Death of the snails was determined by lack of movement, lack of response to gentle prodding with a blunt object, discoloration of the shell, absence of bleeding when they are crushed, and finally by the standard method of immersing the snails in a small amount of 5% sodium hydroxide in a Petri-dish. If bubbles and blood come out of the shell, it is recorded as alive, and Vice-Versa (34, 37 - 39). After 24 hours, LC_50 _values for each extract were calculated according to Litchfield and Wilcoxon (40). The percentage of dead snails was calculated according to Abbot's formula, 1952:

$\mathrm{Killed snails}=\frac{\mathrm{\% Killed by treatment X \% dead in control}}{100-\mathrm{\% dead in control}}$

*Histological preparations*

Treated and control snails were removed from their shells, washed thoroughly with distilled water and fixed in 10% formalin. The snail tissues were processed for paraffin sectioning after embedded in paraplast at 50 ^o^C. The 7 µm sections were stained with iron haematoxylin and eosin, and examined for tissue changes with light microscopy.

## Results and Discussion

*Effect of the tested extracts on the snails*

In Tables 1, 2, 3 and 4, the results of the effect of different *C. viminalis* extracts are listed. LC_25 _and LC_50 _values for each extract were listed in Table 5. Death of snails began after 24 hours from application at low concentrations. In the control experiment, one snail was died after 24 hours, while with the tested extracts at least 8 snails were died. This indicates the molluscicidal activity of the titled plant. Complete death of snails was observed after the third day with the extracts of bark and leaves at concentrations 20 and 40 ppm, while a concentration 10 ppm afforded 66.7% and 63.3% mortality for the extracts of bark and leaves respectively. On the other hand, complete death of snails was observed after the first day with the fruits extract even at a concentration 10 ppm. LC_50 _values for *C. viminalis *fruits bark and leaves were 6.2, 32 and 40 ppm respectively. Thus, *C. viminalis* fruits extract has the highest potential to kill the snails at low concentration and in short periods. According to the World Health Organization of plant molluscicide, screening must kill snails after 24 hours in a concentration 100 ppm or less at constant water temperature (39).

**Table 1:** Death of snails in the control experiment

**Time (day)**	**Number of snails**	**Control**	
		**Death**	**%**
1	30	1	3.3
2	30	2	6.7
3	30	2	6.7
4	30	3	10
5	30	3	10

**Table 2:** Effect of C. *viminalis* fruits extract on B. *alexandrina* snails (number 30).

**Time (day)**	**Number of snails**	**5 ppm**		**10 ppm**		**20 ppm**		**40 ppm**	
		**Death**	**%**	**Death**	**%**	**Death**	**%**	**Death**	**%**
1	30	10	33.3	30	100	30	100	30	100
2	30	17	56.7	30	100	30	100	30	100
3	30	21	70	30	100	30	100	30	100
4	30	26	86.7	30	100	30	100	30	100
5	30	29	96.7	30	100	30	100	30	100

**Table 3:** Effect of *Callistemon viminalis* bark extract on *Biomphalaria alexandrina* snails (number 30).

**Time (day)**	**Number of snails**	**10 ppm**		**20 ppm**		**40 ppm**	
		**Death**	**%**	**Death**	**%**	**Death**	**%**
1	30	9	30	12	40	17	56.66
2	30	16	53.3	23	76.7	25	83.3
3	30	20	66.7	30	100	30	100
4	30	25	83.3	30	100	30	100
5	30	28	93.3	30	100	30	100

**Table 4:** Effect of C. *viminalis* leaves extract on B. *alexandrina* snails (number 30).

**Time (day)**	**Number of snails**	**10 ppm**		**20 ppm**		**40 ppm**	
		**Death**	**%**	**Death**	**%**	**Death**	**%**
1	30	8	26.7	13	43.3	15	50
2	30	15	50	21	70	24	80
3	30	19	63.3	30	100	30	100
4	30	23	76.7	30	100	30	100
5	30	27	90	30	100	30	100

**Table 5:** LC50 values for C. *viminalis* extracts against B. *alexandrina* snails

***C. viminalis*** ** extract**	**LC**_50_
Fruits	6.2
Bark	32
Leaves	40

In the control test, 3 snails out of 30 were dead during the period of the experiment (10%). These results are in agreement with the results of Youssif *et al.* (41) who reported that the daily mortality of *B. alexandrina* was 2.2%.

*Histopathological changes*

Treatment of snails with *C. viminalis* fruits, leaves and bark extracts showed great histopathological signs to the hermaphrodite gland and the digestive tract of the snails. The harmful histopathological changes were a function of each extract concentrations. Treated *B. alexandrina* with a concentration 40 ppm of *C. viminalis* fruits extract showed large vacuoles and degeneration in the hermaphrodite gland, destruction in the follicular membrane and the mature ovum showed losing of the nucleolus (Figure 2). 

**Figure 1:**
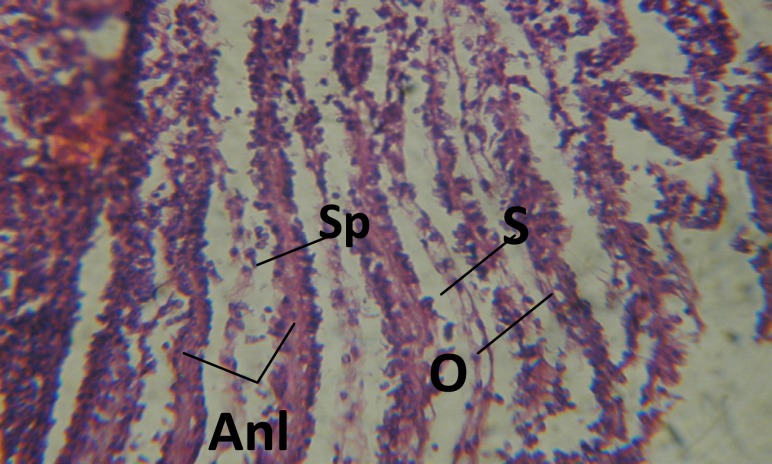
T.S. in control B. *alexandrina* (Hermaphrodite region). Anl= ancel's layer Sp= sperms S= spermatocytes O= oocyte X= 200

**Figure 2:**
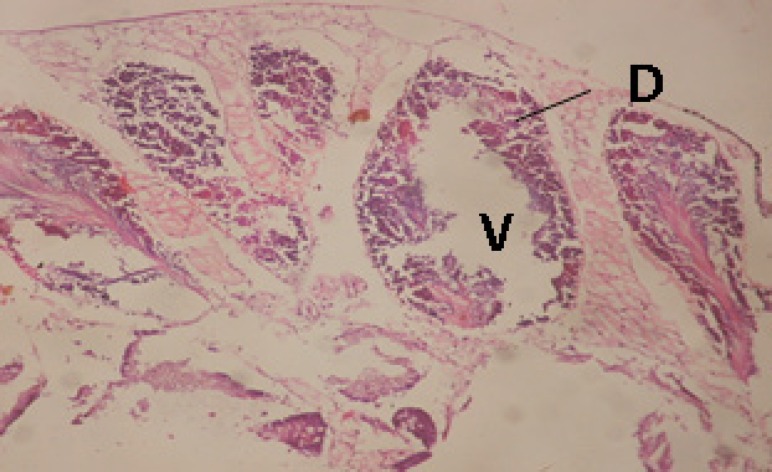
T.S. in treated B. *alexandrina* with 40 ppm fruits extract (Hermaphrodite region). D: degeneration V: vacuoles X= 200

**Figure 3:**
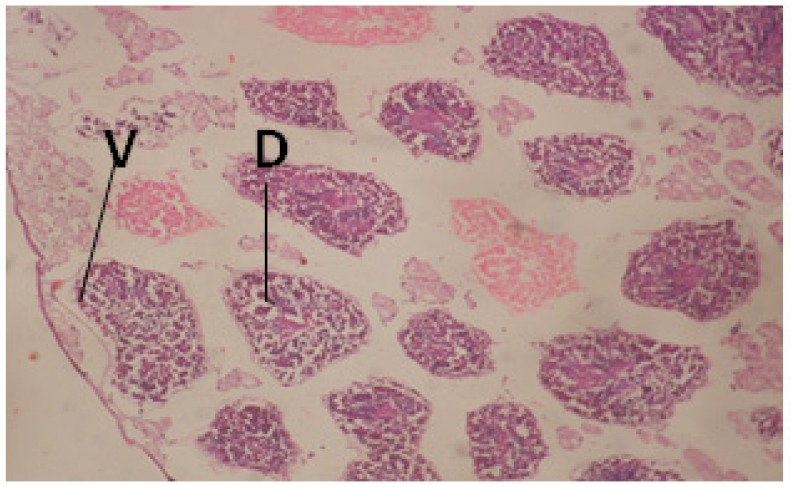
T.S. in treated B. *alexandrina* with 20 ppm fruits extract (Hermaphrodite region). D: degeneration V: vacuoles X= 200

**Figure 4:**
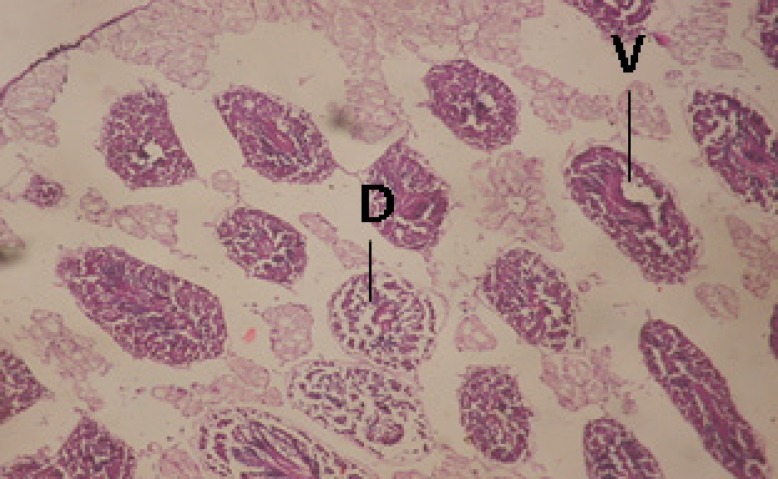
T.S. in treated B. *alexandrina* with 10 ppm fruits extract (Hermaphrodite region). D: degeneration V: vacuoles X= 200

**Figure 5:**
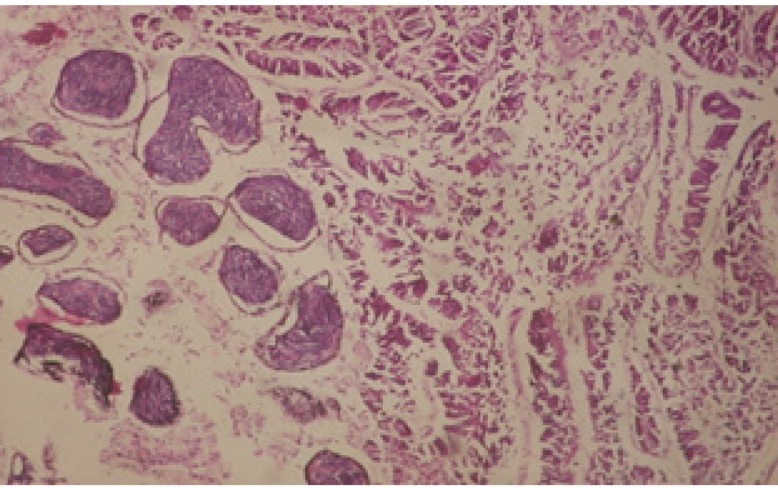
T.S. in treated B. *alexandrina* with 5 ppm fruits extract (Hermaphrodite region). X=200.

**Figure 6:**
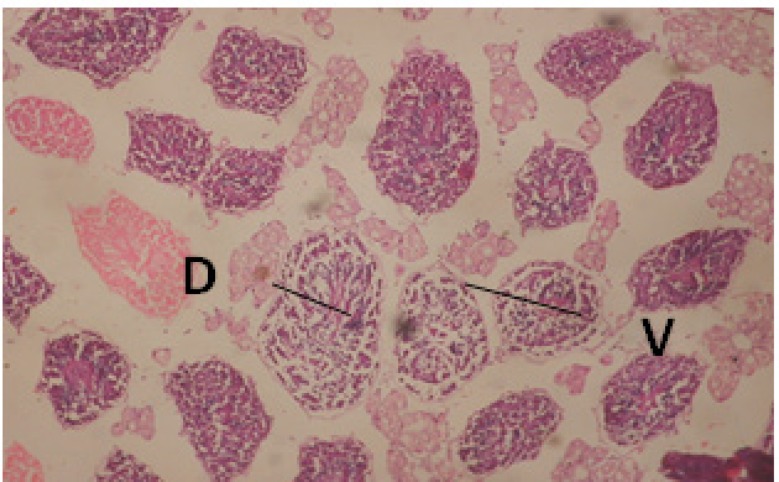
T.S. in treated B. *alexandrina* with 40 ppm bark extract (Hermaphrodite region). D: degeneration V: vacuoles X= 200.

**Figure 7:**
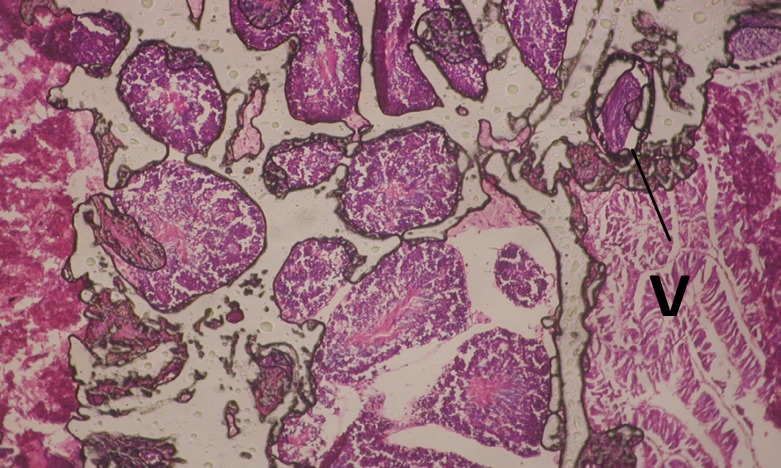
T.S. in treated B. *alexandrina* with 20 ppm bark extract (Hermaphrodite region). V: vacules X= 200.

**Figure 8:**
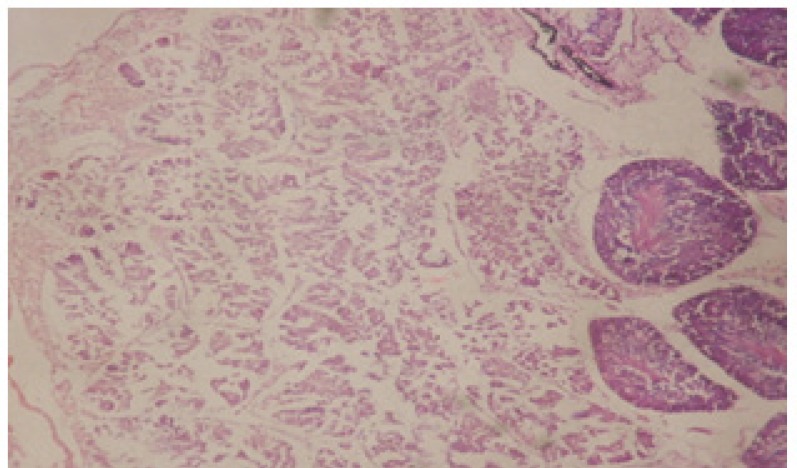
T.S. in treated B. *alexandrina* with 10 ppm bark extract (Hermaphrodite region). X= 200.

**Figure 9:**
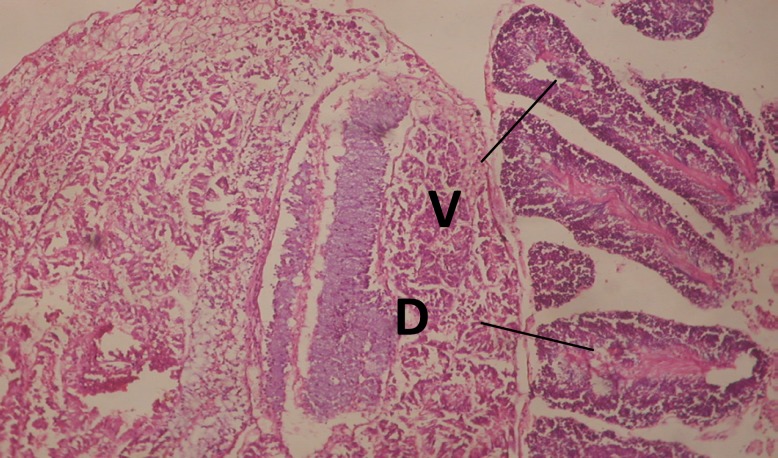
T.S. in treated B. *alexandrina* with 40 ppm leaves extract (Hermaphrodite region). D: degeneration V: vacuoles X= 200.

**Figure 10:**
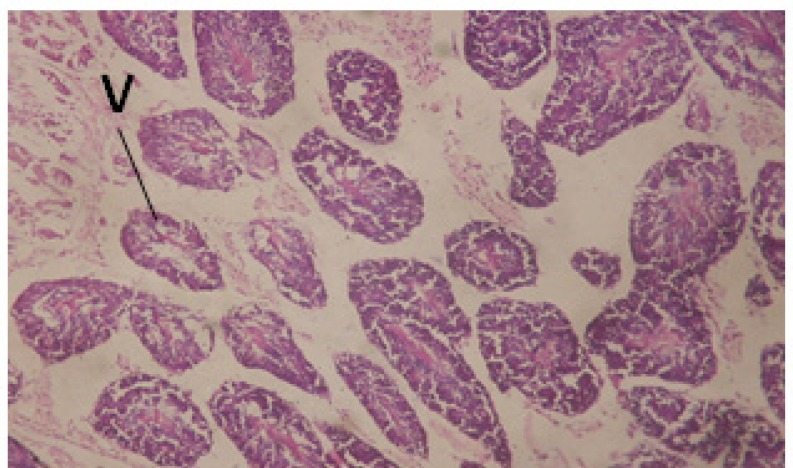
T.S. in treated B. *alexandrina* with 20 ppm leaf extract (Hermaphrodite region). V: vacuoles X= 200.

**Figure 11:**
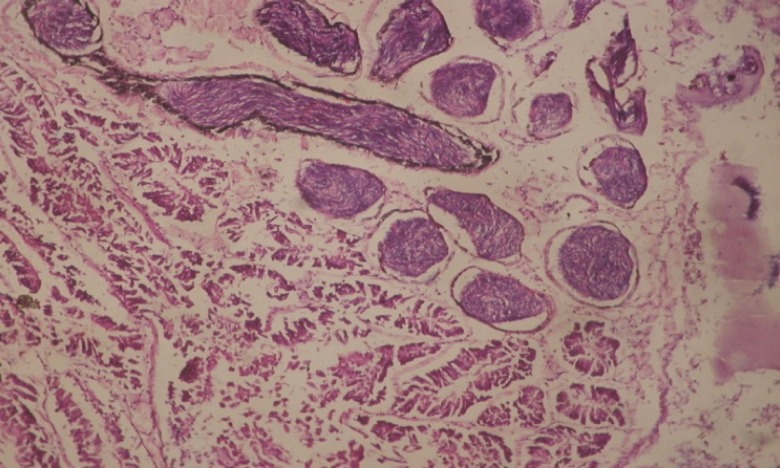
T.S. in treated B. *alexandrina* with 10 ppm leaf extract (Hermaphrodite region). X= 200.

Large vacuoles and great destruction was observed in the digestive acini and the columnar epithelial cells (Figure 13). In the digestive epithelia, there observed large evacuated epithelial cells (Figure 24). While treatment with concentrations 20 and 10 ppm of *C. viminalis* fruits extract showed small vacuoles and little degeneration in the hermaphrodite gland (Figure 3 and 4). The size of vacuoles was not affected by the decreasing in concentrations of the extract and the destruction in the digestive acini and the columnar epithelial cells was less affected (Figure 14 and 15). The evacuation of the epithelial cells in the digestive epithelia was moderate (Figure 25 and 26). On the other hand, treatment with concentration 5 ppm of *C. viminalis* fruits extract showed small vacuoles in the hermaphrodite gland (Figure 5), normal digestive acini and normal columnar epithelial cells (Figure 16) and normal digestive epithelia (Figure 27). This indicates that mild toxicity afforded with the low concentration of the fruit extract. Treated *B. alexandrina* with a concentration 40 ppm of both *C. viminalis* bark and leaves extracts showed small vacuoles and moderate degeneration in the hermaphrodite gland (Figures 6 and 9), small vacuoles in the digestive acini and the columnar epithelial cells (Figures 17 and 20) and small evacuated epithelial cells in the digestive epithelia (Figures 28 and 31) while treatment with concentrations 20 and 10 ppm of both *C. viminalis* bark and leaves extracts was comparable with that of 5 ppm of the fruits extract; small vacuoles in the hermaphrodite gland (Figures 7, 8, 10 and 11), normal digestive acini and normal columnar epithelial cells (Figures 18, 19, 21 and 22) and normal digestive epithelia (Figures 29, 30, 32 and 33).

**Figure 12:**
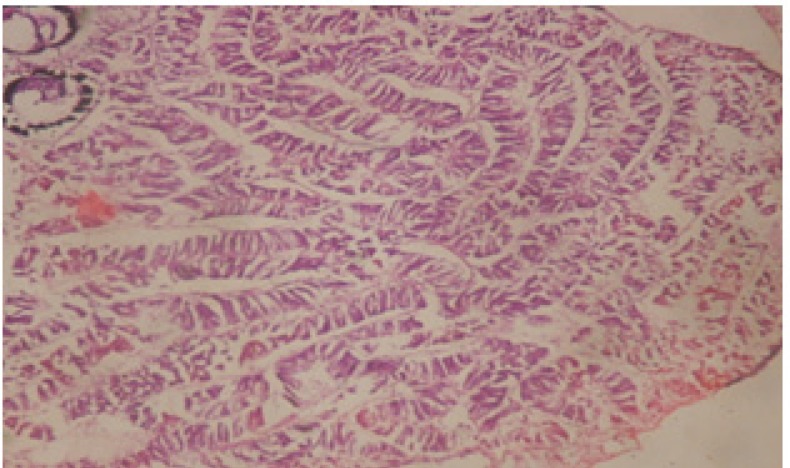
T.S. in control B. *alexandrina* (digestive acini) showing normal columnar epithelial cells. X= 200.

**Figure 13:**
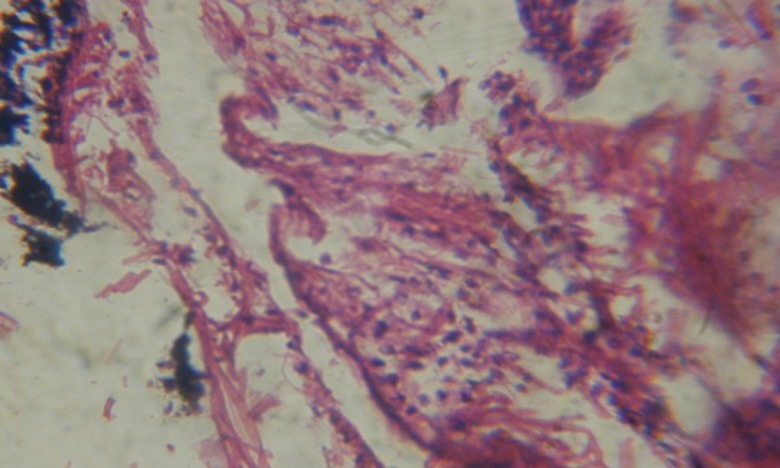
T.S. in treated B. *alexandrina* with 40 ppm fruits extract (digestive acini). X= 200

**Figure 14:**
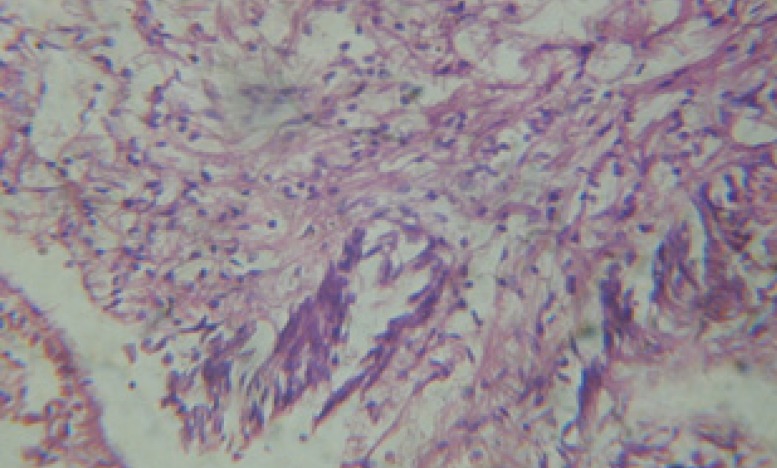
T.S. in treated B. *alexandrina* with 20 ppm fruits extract (digestive acini). X= 200

**Figure 15:**
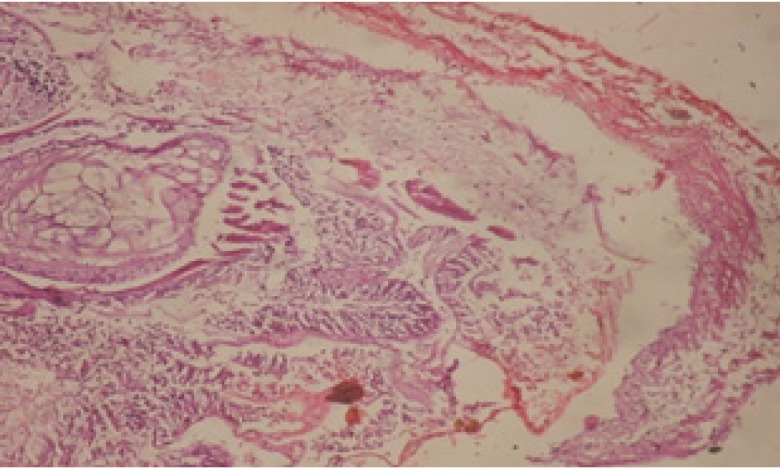
T.S. in treated B. *alexandrina* with 10 ppm fruits extract (digestive acini). X= 200.

**Figure 16:**
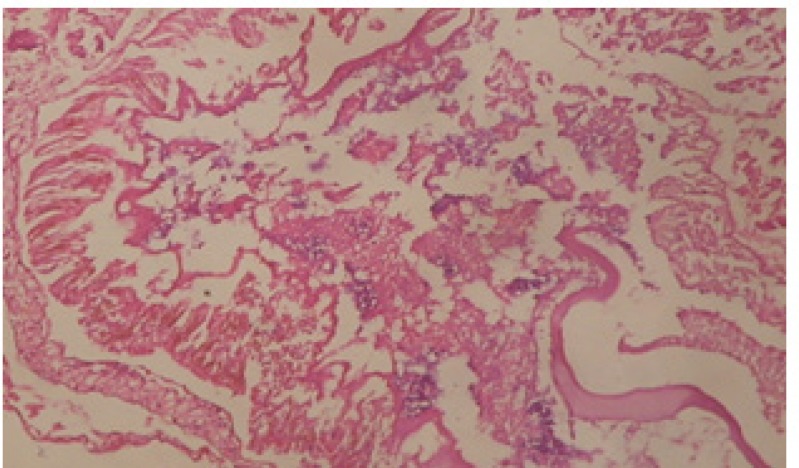
T.S. in treated B. *alexandrina* with 5 ppm fruits extract (digestive acini). X=200.

**Figure 17:**
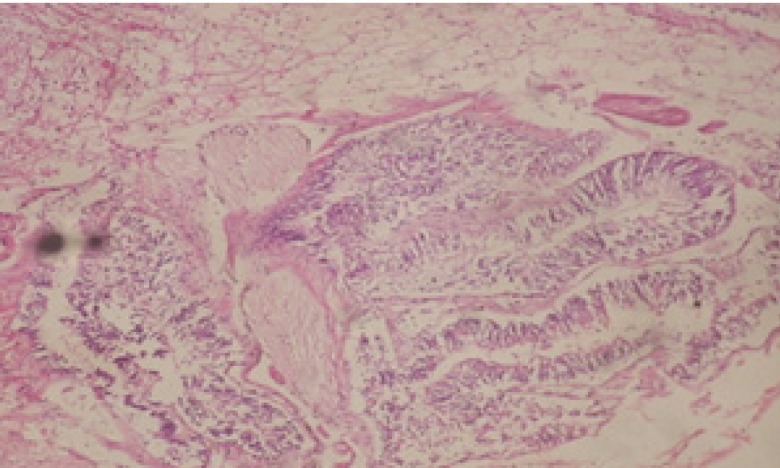
T.S. in treated B. *alexandrina* with 40 ppm bark extract (digestive acini). X= 200

**Figure 18:**
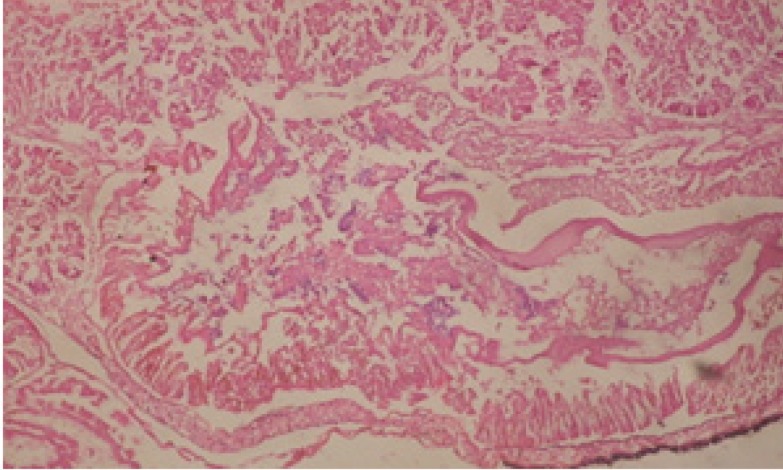
T.S. in treated B. *alexandrina* with 20 ppm bark extract (digestive acini). X= 200.

**Figure 19:**
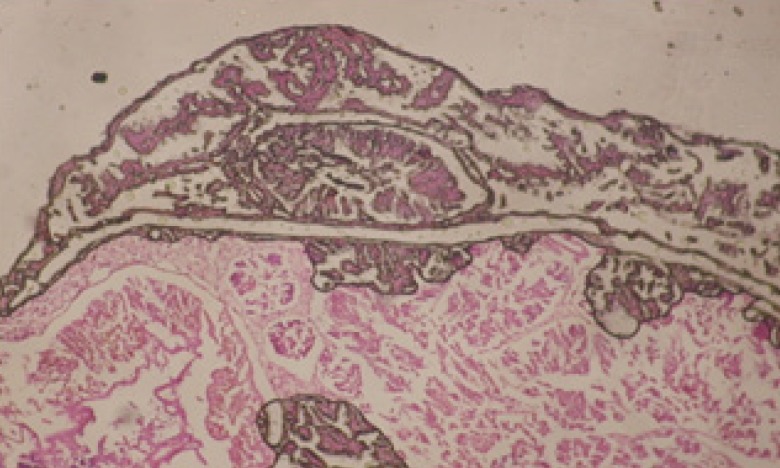
T.S. in treated B. *alexandrina* with 10 ppm bark extract (digestive acini). X= 200.

**Figure 20:**
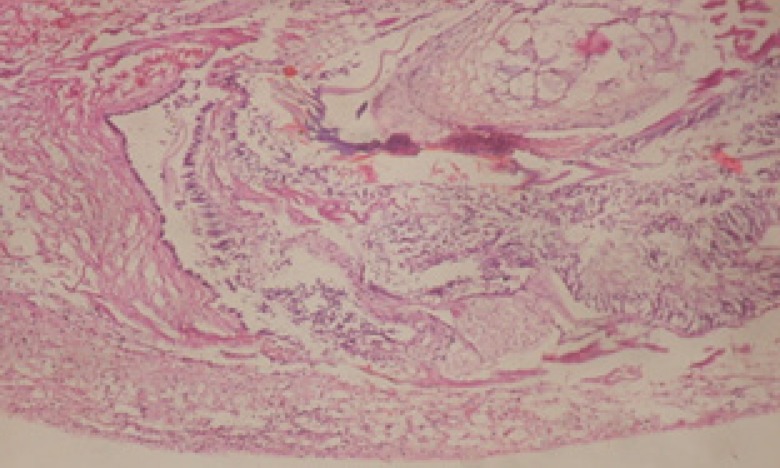
T.S. in treated B. *alexandrina* with 40 ppm leaves extract (digestive acini). X= 200

**Figure 21:**
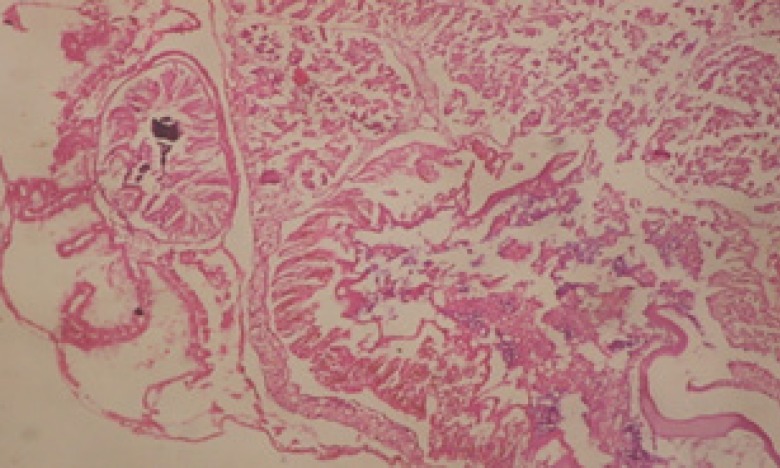
T.S. in treated B. *alexandrina* with 20 ppm leaves extract (digestive acini). X= 200

**Figure 22:**
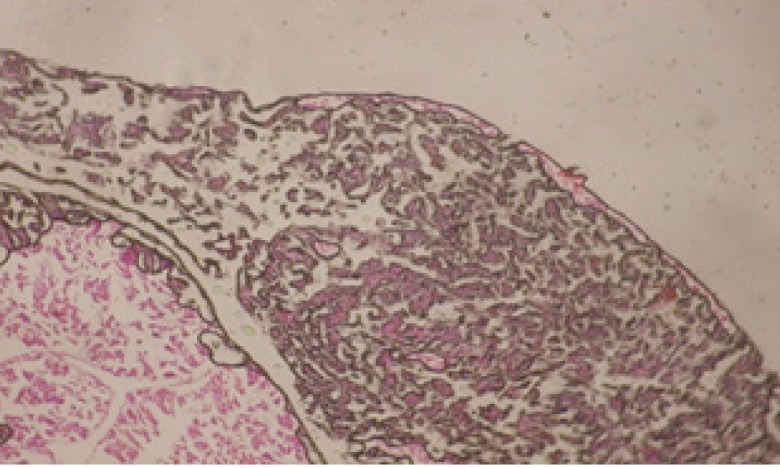
T.S. in treated B. *alexandrina* with 10 ppm leaves extract (digestive acini). X= 200

**Figure 23:**
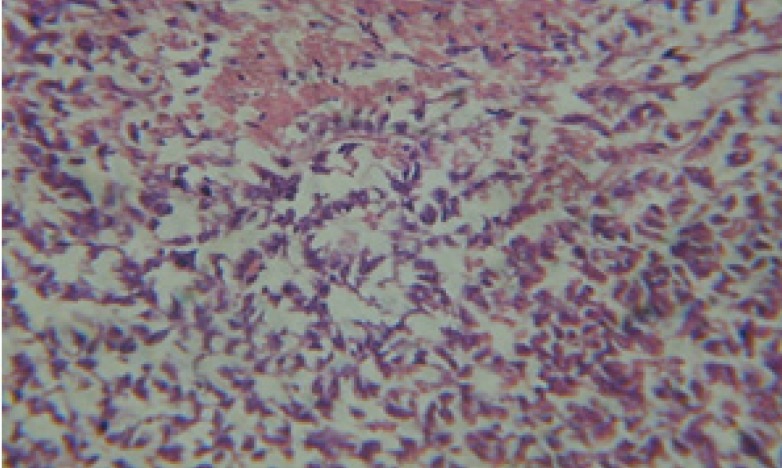
T.S. in control B. *alexandrina* showing digestive epithelia. X = 200

**Figure 24:**
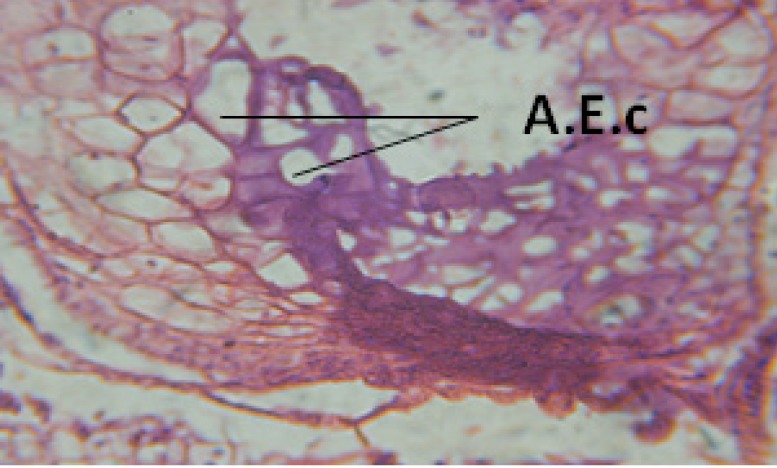
T.S. in treated B. *alexandrina* with 40 ppm fruits extract showing digestive epithelia. A.E.c: evacuated epithelial cells X = 200

**Figure 25:**
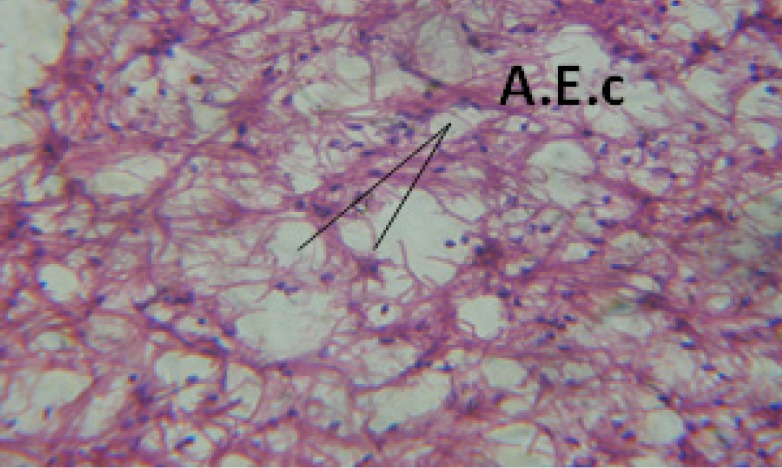
T.S. in treated B. *alexandrina* with 20 ppm fruits extract showing digestive epithelia. A.E.c: evacuated epithelial cells X = 200

**Figure 26:**
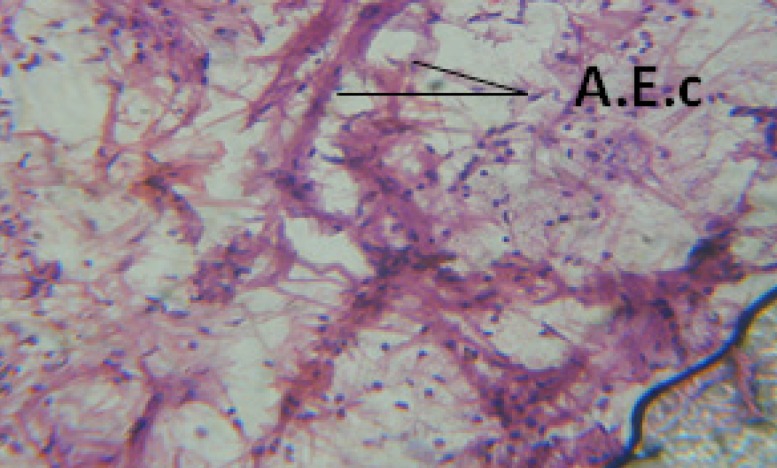
T.S. in treated B. *alexandrina* with 10 ppm fruits extract showing digestive epithelia. A.E.c: evacuated epithelial cells X = 200

**Figure 27:**
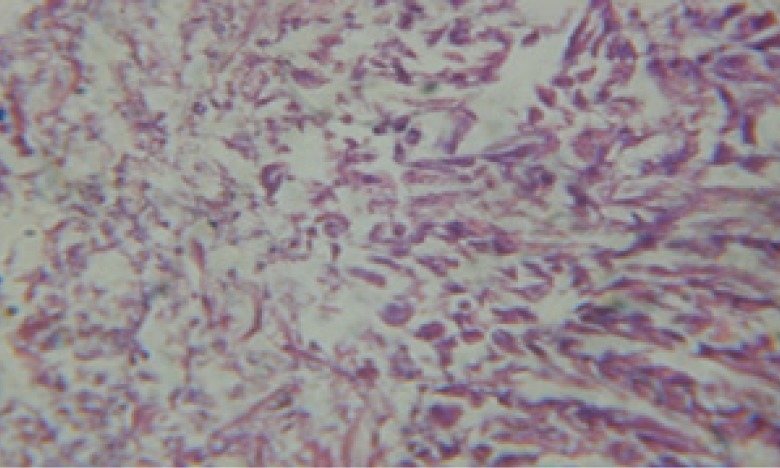
T.S. in treated B. *alexandrina* with 5 ppm fruits extract showing digestive epithelia. A.E.c: evacuated epithelial cells X = 200

**Figure 28:**
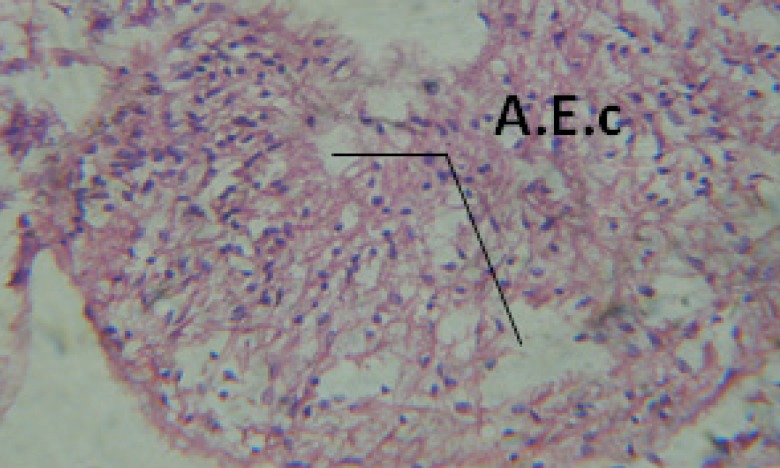
T.S. in treated B. *alexandrina* with 40 ppm bark extract showing digestive epithelia. A.E.c: evacuated epithelial cells X = 200

**Figure 29:**
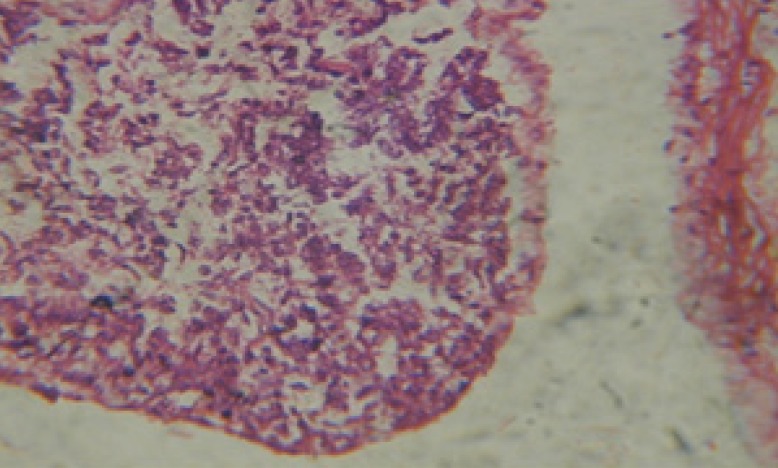
T.S. in treated B. alexandrina with 20 ppm bark extract showing digestive epithelia. X = 200.

**Figure 30:**
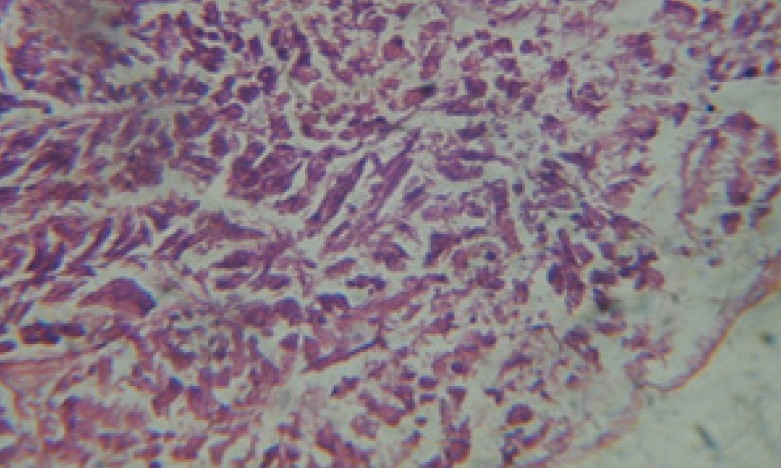
T.S. in treated B. *alexandrina* with 10 ppm bark extract showing digestive epithelia. X = 200

**Figure 31:**
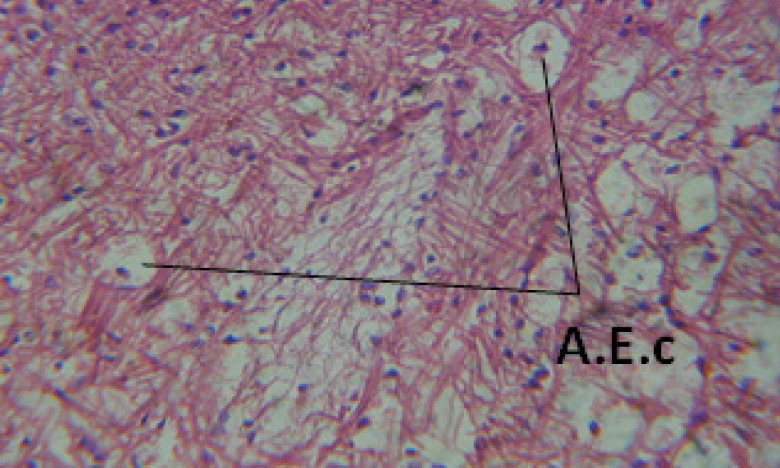
T.S. in treated B. *alexandrina* with 40 ppm leaves extract showing digestive epithelia. A.E.c: evacuated epithelial cells X = 200

**Figure 32:**
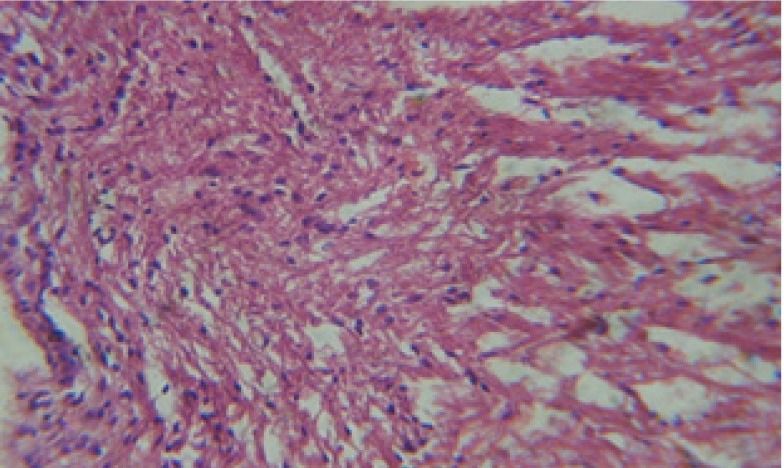
T.S. in treated B. *alexandrina* with 20 ppm leaves extract showing digestive epithelia. X = 200

**Figure 33:**
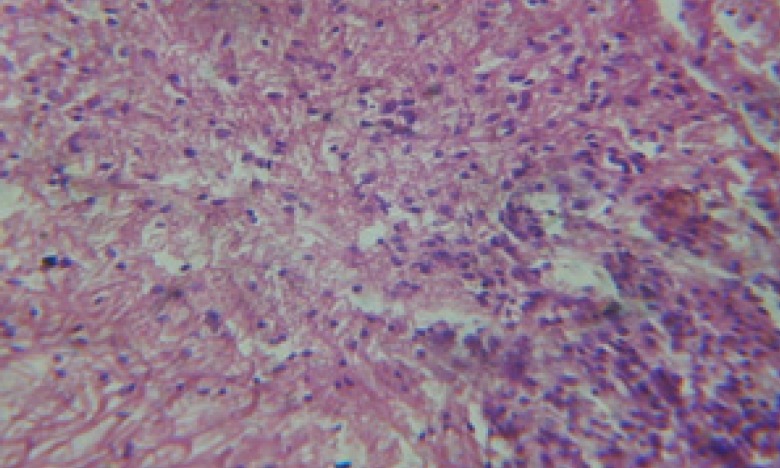
T.S. in treated B. *alexandrina* with 10 ppm leaves extract showing digestive epithelia. X = 200

The tested plant extracts proved positive molluscicidal activity against *B. alexandrina* snails. It seems that the target tissues, for the tested extracts, were the hermaphrodite gland and the digestive tract. Destruction of the epithelial layer, vaculation and degeneration of secretory cells are the histopathological signs detected after treatment with the tested extracts.
